# Recruitment and retention rates in randomised controlled trials of exercise therapy in people with multimorbidity: a systematic review and meta-analysis

**DOI:** 10.1186/s13063-021-05346-x

**Published:** 2021-06-14

**Authors:** Lasse K. Harris, Søren T. Skou, Carsten B. Juhl, Madalina Jäger, Alessio Bricca

**Affiliations:** 1 Research Unit for Musculoskeletal Function and Physiotherapy, Department of Sports Science and Clinical Biomechanics, University of Southern Denmark, 5230 Odense M, Denmark; 2 Department of Physiotherapy and Occupational Therapy, Næstved-Slagelse-Ringsted Hospitals, Region Zealand, 4200 Slagelse, Denmark; 3 Department of Physiotherapy and Occupational Therapy, University Hospital of Copenhagen Herlev and Gentofte, Copenhagen, Denmark

**Keywords:** Multimorbidity, Recruitment, Retention, Exercise therapy, Randomised controlled trial, Systematic review

## Abstract

**Aim:**

To quantify recruitment, retention and differential retention rates and associated trial, participant and intervention characteristics in randomised controlled trials (RCTs) evaluating the effect of exercise therapy in people with multimorbidity.

**Data sources:**

MEDLINE, EMBASE, CINAHL and CENTRAL from 1990 to April 20, 2020.

**Study selection:**

RCTs including people with multimorbidity comparing exercise therapy with a non-exposed comparator group reporting at least one of the following outcomes: physical function, health-related quality of life, depression symptoms, or anxiety symptoms.

**Data extraction and synthesis:**

Recruitment rates (proportion of people randomised/proportion of people eligible), retention rates (proportion of people providing the outcomes of interest/proportion randomised) and differential retention rates (difference in proportion of people providing the outcomes in the intervention group and comparator group) were calculated. Meta-analysis using a random-effects model was used to estimate pooled proportions. Methodological quality was assessed using Cochrane ´Risk of Bias tool 2.0´ for individual studies, and the GRADE approach was used to assess the overall quality of the evidence.

**Results:**

Twenty-three RCTs with 3363 people were included. The pooled prevalence for recruitment rate was 75% (95%CI 66 to 84%). The pooled prevalence for retention rate was 90% (95%CI 86 to 94%) at the end of the intervention (12 weeks; interquartile range (IQR) (12 to 12)). Meta-regression analyses showed that increasing age and including a higher proportion of people with hypertension was associated with lower retention rates. Retention rates did not differ between the intervention and comparator groups. The overall quality of the evidence was deemed very low.

**Conclusion:**

Three in four eligible people with multimorbidity were randomised to RCTs using exercise therapy, of which nine out of 10 provided end of treatment outcomes with no difference seen between the intervention and comparison groups. However, the results must be interpreted with caution due to large differences between the included studies.

**Trial registration:**

ClinicalTrials.govCRD42020161329. Registered on 28 April 2020.

**Supplementary Information:**

The online version contains supplementary material available at 10.1186/s13063-021-05346-x.

## Background

Multimorbidity, defined as the coexistence of two or more chronic conditions, is a major priority in health care and research [1, 2]. A possible explanation is that multimorbidity is becoming a rapidly escalating problem in most healthcare systems because of its increasing prevalence with age and association with increased mortality, worse functional status and reduced health-related quality of life (HRQoL) [2–5]. This increasing burden combined with the complexity of multimorbidity challenges the current perspectives of standard care, which focus on single disease-oriented management programs rather than specific patient-oriented care [6, 7].

Chronic conditions such as osteoarthritis, hypertension, type 2 diabetes mellitus, depression, heart failure, ischemic heart disease, and chronic obstructive pulmonary disease are among the leading causes of global disability, affecting hundreds of millions of people worldwide [8]. These conditions often coexist and are linked by a common risk factor (physical inactivity) and pathogenesis (systemic low-grade inflammation) which potentially causes a cascade of reactions resulting in the development of a ‘vicious cycle’ of chronic diseases and poor outcomes [9, 10].

Randomised controlled trials (RCTs) are the gold standard of experimental study designs [11]. However, RCTs with poor recruitment and retention rates are considered a threat to the validity of the results [12] and it is widely agreed that research that identifies strategies for improving recruitment and retention is a priority [13]. Prior systematic reviews within the medical field have reported wide ranges of recruitment and retention rates [14–17], and individual studies have identified that recruiting and retaining patients in multimorbidity in clinical trials is challenging [18, 19]. Possible sources of poor recruitment and retention include lack of good communication between the patient and recruitment staff and negative attitude of research staff [20].

Exercise therapy appears to be a safe and effective treatment for people with multimorbidity [21]; however, a comprehensive summary of recruitment and retention rates in people with multimorbidity participating in RCTs of exercise therapy is lacking. Evaluating recruitment and retention rates, identifying strategies to improve recruitment and retention, and determining if retention between exercise and control groups are different in exercise therapy RCTs would help in the design and conduct of future RCTs for people with multimorbidity by providing a realistic perspective on crucial parts of the RCT beneficial for both clinical and research practise. Therefore, we investigated the recruitment, retention and differential retention rates of people with multimorbidity participating in RCTs evaluating the effect of exercise therapy. We also examined trial, participant and intervention characteristics associated with improved recruitment, retention and differential retention.

## Methods

### Protocol and registration

This systematic review was reported according to the Preferred Reporting Items for Systematic Reviews and Meta-analyses guidelines (PRISMA Checklist: Additional file 1) [22] and was based on a protocol with pre-specified study selection, eligibility criteria, data extraction and strategy for data synthesis [23] in accordance with the Cochrane Handbook for Systematic Reviews of Interventions [24]. The protocol was registered at PROSPERO (CRD42020161329) and was also made publicly available via the Open Science Framework website [25, 26] before completion of the title/abstract screening phase.

### Information sources

We used the same search strategy developed from our previous systematic review which investigated the effect of exercise therapy in people with multimorbidity [23]. Information was retrieved from the following sources:

- Searching MEDLINE via PubMed, EMBASE via Ovid, CINAHL (including preCINAHL) via EBSCO and the Cochrane Central Register of Controlled Trials (CENTRAL) up to October 12, 2019, with no restriction on language. Only RCTs published since 1990 were included as the reporting and treatment of multimorbidity have changed considerably in recent years. Searches were repeated for the period from October 2019 to April 20, 2020, in the same databases to identify additional studies published before manuscript submission.
- Screening the reference lists of the latest Cochrane reviews investigating the effect of therapeutic exercise on the following conditions: osteoarthritis, hypertension, diabetes type 2, depression, heart disease or heart failure, and chronic obstructive pulmonary disease.
- Screening the reference lists of included RCTs.
- Screening The World Health Organization’s International Clinical Trials Registry Platform (ICTRP) http://apps.who.int/trialsearch/ which comprise the 16 primary registries of the WHO registry network and ClinicalTrials.gov.
- Web of Science (WoS) was used for citation tracking by searching studies citing the RCTs included in this systematic review.

The following constructs were used for the literature search in MEDLINE via PubMed: osteoarthritis, co-existing health problem, diabetes mellitus, depression, hypertension, pulmonary disease, chronic obstructive, myocardial ischemia, exercise and randomised controlled trial. They were combined with the Boolean operators OR/AND, searched as Title/Abstract (i.e., TIAB), and as keywords Medical Subject Headings (i.e., MeSH). The detailed search strategy in MEDLINE (https://osf.io/84vzn/) was made publicly available at Open Science Framework [26] and was adjusted to fit the other databases.

### Eligibility criteria

#### Study design

English language RCTs published in peer-reviewed journals or unpublished RCTs from registries with available and relevant data.

#### Type of participants

Studies including at least 80% of the people with at least two of the following conditions: osteoarthritis of the hip or knee, heart failure, ischemic heart disease, hypertension (systolic blood pressure >90 and diastolic blood pressure >140), type 2 diabetes mellitus, chronic obstructive pulmonary disease and depression as defined by the studies or calculated based on baseline participants characteristics. This pragmatic approach was pre-specified and adopted to capture all the studies which included people with multimorbidity, given the expected inconsistency of reporting of the conditions across trials. Studies including children and adolescents (i.e., mean age <18 years) were excluded.

#### Types of intervention

Studies which included exercise therapy interventions with or without additional pharmacotherapy or other adjuvant interventions (e.g. weight loss) were eligible for inclusion. Exercise therapy is defined as ‘a regimen or plan of physical activities designed and prescribed for specific therapeutic goals with the purpose of restoring normal physical function or to reduce symptoms caused by diseases or injuries’ [27]. Intervention arms delivering unstructured exercise programs (e.g. providing a pedometer or booklet to the people without a specific plan for physical activity) were excluded.

#### Type of outcomes of the individual studies

Studies assessing at least one of the following outcomes were eligible for inclusion:

- Physical outcome: Objectively measured and self-reported physical function (e.g. 6-min walking test, 36-item Short-Form Health Survey (SF-36))
- Psychosocial outcome: HRQoL (e.g. EQ-5D questionnaire), depression symptoms or anxiety symptoms (e.g. Hospital Anxiety and Depression Scale)

The rationale for including these outcomes is based on a consensus study that identified outcomes for multimorbidity intervention studies [28] and the fact that they are generic and widely used across the conditions of interest. Additionally, to avoid multiplicity, we used a hierarchy of selection rules for the outcomes described elsewhere [23].

The primary outcome measures of this systematic review were as follows:

- Recruitment rates: Proportion of eligible people recruited (proportion of people randomised/proportion of people eligible). The proportion of people eligible included those saying no to being included.
- Retention rates: Proportion of randomised people (proportion of people providing the outcomes of interest/proportion randomised) providing physical (i.e. physical function) and/or psychosocial outcomes (i.e. HRQoL, depression symptoms and anxiety symptoms) at the end of the intervention and the follow-up closest to 12 months.
- Differential retention rates: Difference in proportion of people providing physical (i.e. physical function) and/or psychosocial outcomes (i.e. HRQoL, depression symptoms and anxiety symptoms) in the intervention and comparator group, at the end of the intervention and the follow-up closest to 12 months.

### Study selection

The identified studies from the literature search were uploaded to EndNote X9. Two reviewers (LKH and AB) independently screened titles and abstracts, and all studies deemed eligible by at least one of the reviewers were checked independently in full text. Disagreement between the reviewers in inclusion was discussed until consensus was reached. If consensus could not be reached, a third author’s opinion (CBJ) was sought to achieve consensus. We checked whether multiple reports from the same study were published by juxtaposing author names, treatment comparisons, sample sizes or outcomes. If multiple reports of the same studies provided different study characteristics (e.g. number of people and presence of comorbidities), we contacted the authors for clarifications.

### Risk of bias and overall quality assessment of the evidence

Two reviewers (LKH and AB) independently assessed the methodological quality of the included studies using the Cochrane ‘Risk of Bias Tool 2.0’ [24]. The Risk of Bias Tool was applied because all the included studies were effect estimation studies. Poor methodology in the studies therefore influence recruitment and retention rates. Bias was assessed in five distinct domains: bias arising from the randomisation process, bias due to deviations from intended interventions, bias due to missing outcome data, bias in the measurement of the outcome (blinding) and bias in the selection of the reported result. Within each domain, the two reviewers answered one or more signalling questions (e.g. Was the allocation sequence random? Were people aware of their assigned intervention during the trial?) which led to judgements of ‘low risk of bias’, ‘some concerns’, or ‘high risk of bias’. The judgements within each domain led to an overall risk-of-bias judgement for the result being assessed.

The overall quality of evidence for the estimates was evaluated by two reviewers (LKH and AB) using the Grading of Recommendations Assessment, Development and Evaluation (GRADE) approach [29]. GRADE is a systematic approach to rate the quality of evidence across studies for specific outcomes. It is based on five domains that involve the methodological flaws of the studies (i.e. risk of bias), the heterogeneity of results across studies (i.e. inconsistency), the generalizability of the findings to the target population (i.e. indirectness), the precision of the estimates (i.e. imprecision) and the risk of publication bias.

### Data collection process

Our data extraction sheet was developed based on the Cochrane Collaboration data collection form for intervention reviews: RCTs only [30] and are available at open science framework [26]. Thereafter, pilot testing was performed using three of the included RCTs randomly chosen to refine the data extraction sheet before extracting data from all the included studies. Two reviewers (LKH and AB) performed data extraction for all included studies.

### Data extraction

All data were extracted at a study level (e.g. we evaluated whether age was associated with increased/reduced recruitment rates across studies). To calculate recruitment rates, we extracted the number of people randomised and the number of people eligible. Similarly, to calculate retention and differential retention rates, we extracted the number of people providing outcomes in the intervention and comparator groups, at end of the intervention and closest to 12-month follow-up. Additionally, we extracted the following data to investigate the impact of the study, intervention, comparator and outcomes characteristics on the outcomes of interest.

#### Trial characteristics

Trial design (e.g. factorial, open design), country and clinical location (in case of multilocation studies, primary investigator affiliation applied), recruitment strategy used (e.g. one-to-one, news advertisement, online) and retention strategy used (e.g. financial incentives, phone reminders), recruitment strategy length (in months), the total number of people assessed for eligibility, location of the recruitment (e.g. hospital, community of GP practice), patient public involvement (people involved in the intervention development), eligibility assessment strategy (e.g. via registry, database, in person, via phone call pre-screening) and reasons for people to dropout.

#### Participant characteristics

Age, % female, body mass index (BMI), socioeconomic status (labelled as `low SES` when the majority of people are described as having low education levels, low income, being unemployed or sample otherwise labelled as `low SES`), baseline severity of the conditions and number, and type and severity of other conditions.

#### Intervention and comparator characteristics

Components of intervention (i.e. therapeutic exercise, exercise + diet), type of exercise/comparator intervention (i.e. aerobic, neuromuscular, strengthening or a combination), frequency of the sessions (times per week), intensity of the session (% of maximum pulse, or % of 1 repetition maximum), volume of the sessions, mode of delivery (i.e. one-to-one, group or self-help) setting (i.e. home-based, clinic-based or a combination), duration of the interventions (in weeks), supervision (i.e. yes, no or a combination), tailoring (i.e. intervention developed according to guidelines and individual people’s needs), and adherence to intervention (i.e. the total number of sessions attended out of the total number of sessions available).

#### Outcome characteristics

Time points assessed and the magnitude of objectively and subjectively measured changes (e.g. change in HRQoL). As previously mentioned, a hierarchy of selection rules for the outcomes was applied. We prioritised data extraction of outcome measures important for the participants [28] and generic over disease-specific measures [23]. For objectively measured physical function, we prioritised (1) the 6-min walking test, (2) incremental shuttle walking test and (3) any other outcome measure related to daily function (e.g. chair stand test). For self-reported physical function, we prioritised outcomes in the following order: (1) the SF-36 physical function subscale, (2) the SF-36 role function subscale and (3) any other self-reported measure of physical function. For HRQoL outcomes, we prioritised (1) the EQ-5D questionnaire and (2) any other HRQoL questionnaires (e.g. The Minnesota living with heart failure questionnaire). For depression symptoms, we prioritised (1) The Beck Depression Inventory (BDI) and (2) any other depression questionnaires (e.g. the Hospital Anxiety and Depression Scale (HADS depression). For anxiety symptoms, we prioritised (1) State Trait Anxiety Inventory questionnaire and (2) any other anxiety questionnaires (e.g. HADS anxiety).

If we were unable to extract the abovementioned data from the included RCTs, we emailed the corresponding author of each study with a checklist of the data we aimed to obtain. If the corresponding author did not reply, we contacted a second author as well for obtaining the information and so forth. After 3 days, we sent a reminder including the last author of the study. After 7 days, a reminder was re-sent to the corresponding and last author. Another reminder followed 10 days later. Finally, we considered the data as missing if no communication from the authors was received 15 days after sending the first email.

### Summary measures and synthesis of results

Recruitment, retention, and differential retention rates of people with multimorbidity were the outcome measures being calculated. Estimates of these rates were pooled using random-effects proportion meta-analyses (Stata V.16.1 metaprop command) [31]. Binomial proportion 95% CIs for individual studies were calculated around study-specific and pooled prevalence based on the score-test statistic. Heterogeneity was examined as between-study variance and calculated as the I-squared statistic measuring the proportion of variation in the combined estimates due to between-study variance [24]. An I-squared value of 0% indicated that no inconsistency existed between the results of individual trials, where an I-squared value of 100% indicated maximal inconsistency.

### Additional analyses

We pre-specified subgroup and meta-regression analyses to explore heterogeneity. Relevant study-level covariates, able to decrease inconsistencies measured as the I-squared statistic (and thus the between-study variance Tau-square), were investigated to explore possible association between study, participants, intervention and comparator group characteristics on recruitment, retention and differential retention rates. In accordance with the Cochrane Handbook, we performed meta-regression analyses when at least 10 studies reported data for the relevant covariates [24].

## Results

### Study selection

**Fig. 1:**
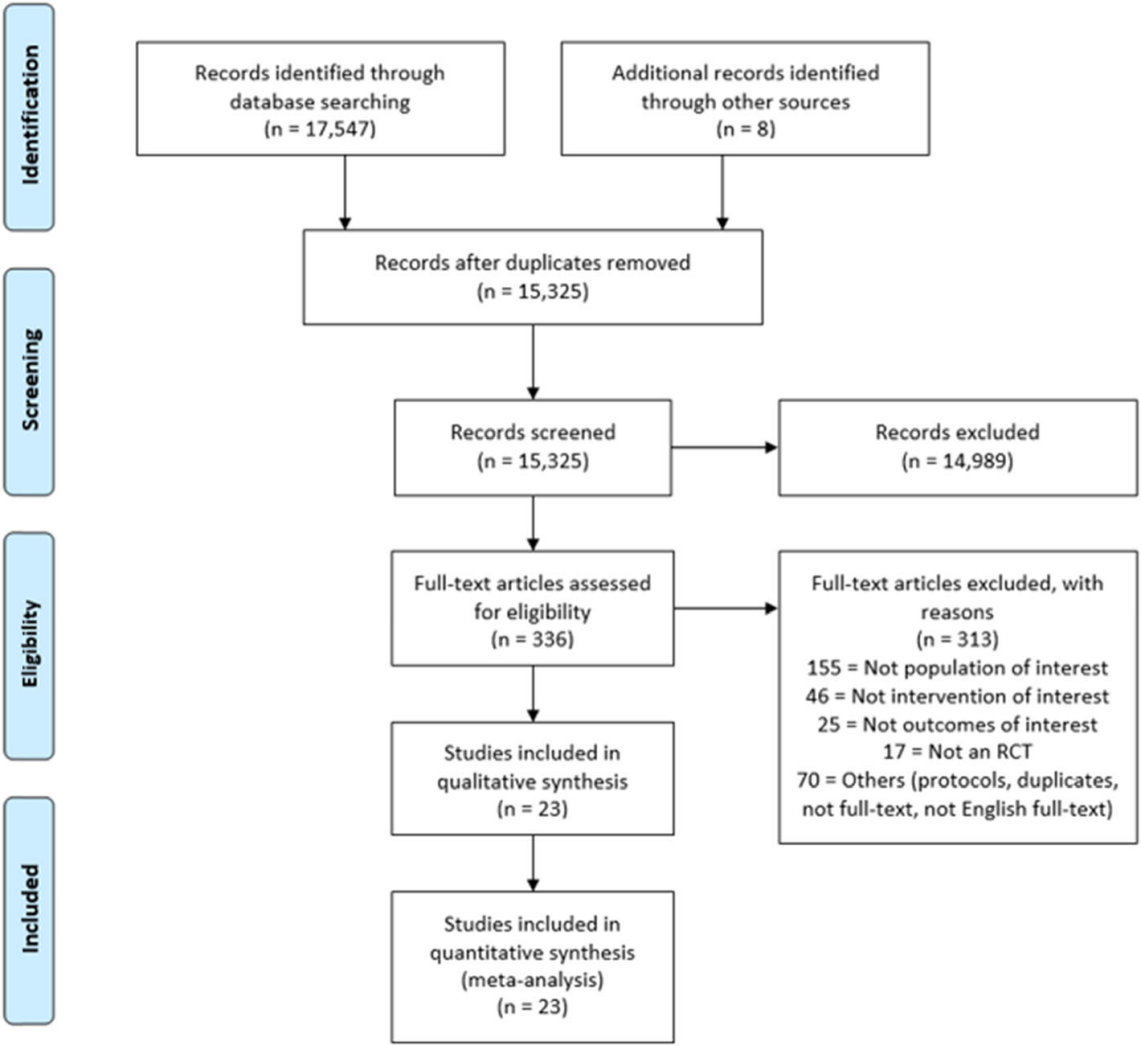
Flow diagram of the included RCTs. RCT = randomised controlled trial

A total of 17,547 studies were identified by the search strategy. After removing duplicates, assessing title and abstracts, and full-text assessment of the remaining studies, we included 23 RCTs published in 24 papers (Fig. 1).

### Study characteristics

Table 1 summarises the characteristics of the included RCTs. The studies were conducted across 18 countries, including Europe [33, 34, 36, 40, 42, 43, 45, 47, 48, 52, 55], USA [32, 35, 37–39, 46, 50, 54], Australia [41], and Asia [44, 49, 51, 53]. A total of 18 studies reported the type of recruitment strategy used with 50% using a mix of both direct (i.e. potential people approached individually) and indirect (i.e. potential people approached, e.g. via news advertisement or flyers) strategies. The recruitment setting was classified as outpatient (k=13) and at hospitals (k=7) with recruitment length varying widely from 2 to 53 months.

**Table 1:** Characteristics of the included studies

Author, publication year	Country, recruitment setting	Recruitment length, strategy used	Intervention length, outcome measures	Proportion of people for each multimorbidity condition, population characteristics
Gary et al. [32]	USA University clinic	18 months Direct and indirect approach^a,b^	12 weeks PF, HRQoL, DEP	KOA 68%, HYP 88%, T2DM 31%, DEP 44%, HF 100%, COPD 34% 32 people, mean age 68 years, 100% female, mean BMI 33.5
Koukouvou et al. [33]	Greece Hospital	2 months Direct and indirect approach^a,b^	26 weeks HRQoL, DEP, ANX	HYP 12%, DEP 100%, HF 100% 26 people, mean age 52 years, 0% female, mean BMI 28.1
Kulcu et al. [34]	Turkey University school	n/a	8 weeks HRQoL, DEP, ANX	DEP 100%, HF 100% 53 people, mean age 59 years, 27% female, n/a
Gary et al. [35]	USA Outpatient clinic	14 months Direct and indirect approach^a,b^	12 weeks PF, HRQoL, DEP	HYP 88%, T2DM 32%, DEP 100%, HF 100% 74 people, mean age 65.8 years, 57% female, n/a
Asa et al. [36]	Sweden n/a	n/a	8 weeks PF, HRQoL, DEP, ANX	T2DM 100%, HF 100% 20 people, mean age 67.4 years, 20% female, mean BMI 29
Blumenthal et al. [37] (UPBEAT)	USA Outpatient clinics	53 months Direct and indirect approach^a,b^	16 weeks DEP	HYP 19%, DEP 100%, HF 100%, 101 people, mean age 63.9 years, 32% female, mean BMI 31
Blumenthal et al. [38] (HF-ACTION)	USA, Canada, France 82 medical centres	47 months Direct approach^a^	12 weeks DEP	HYP 61%, DM* 10%, DEP 100%, HF 100% 653 people, mean age 56 years, 92% female, mean BMI 31.5
Gary et al. [39]	USA Outpatient clinic	6 months Indirect approach^b^	12 weeks PF, HRQoL, DEP	HYP 50%, T2DM 50%, DEP 70%, HF 100% 24 people, mean age 60 years, 50% female, mean BMI 34
Oerkild et al. [40]	Denmark Rehabilitation unit	19 months Direct and indirect approach^a,b^	12 weeks PF, HRQoL, DEP, ANX	HYP 73%, T2DM 23%, DEP 18%, HF 100%, COPD 28% 40 people, mean age 76.9 years, 43% female, mean BMI 27
Leung et al. [41]	Australia Hospital	41 months Direct approach^a^	12 weeks PF, HRQoL, DEP, ANX	KOA 60%, HYP 55%, T2DM 19% IHD 33%, COPD 100% 42 people, mean age 73 years, 36% female, mean BMI 27.4
Nolte et al. [42] and Edelmann et al. [43] (Ex-DHF-P)	Germany 3 University hospitals	7 months n/a	12 weeks PF, HRQoL, DEP	HYP 82%, T2DM 14%, DEP 64%, HF 100% 67 people, mean age 65 years, 56% female, mean BMI 31
Keihani et al. [44]	Iran n/a	n/a	8 weeks PF, DEP, ANX	DEP 100%, HF 100% 65 people, mean age 61.2 years, 40% female, mean BMI 26.1
Pibernik-Okanovic et al. [45]	Croatia Hospital	19 months Indirect approach^b^	6 weeks HRQoL, DEP	T2DM 100%, DEP 100% 209 people, mean age 58.1 years, 54% female, mean BMI 30
Schneider et al. [46]	USA University school	21 months Direct and indirect approach^a,b^	12 weeks DEP	T2DM 100%, DEP 100% 29 people, mean age 53.4, 100% female, mean BMI 34.6
Hinrichs et al. [47] (Homefit)	Germany General practices	14 months Direct and indirect approach^a,b^	12 weeks PF, HRQoL	KOA 60%, HOA 46%, HYP 90%, T2DM 40%, HF 33%, IHD 29%, COPD 22% 209 people, mean age 79.8 years, 74% female, mean BMI 30.7
Bernocchi et al. [48]	Italy Hospital	15 months Direct approach^a^	16 weeks PF, HRQoL	HF 100%, COPD 100% 112 people, mean age 70.5 years, 18% female, mean BMI 28.1
Abdelbasset et al. [49]	Saudi Arabia University hospital	4 months Direct approach^a^	6 weeks DEP	HYP 20%, DEP 100%, HF 100% 69 people, mean age 52.7 years, 28% female, mean BMI 30
de Groot et al. [50] (ACTIVE II)	USA Medical practices	48 months Direct and indirect approach^a,b^	12 weeks PF, HRQoL, DEP	T2DM 100%, DEP 100% 140 people, n/a
Leung et al. [51]	Hong Kong Outpatient clinic	3 months Direct approach^a^	12 weeks PF, HRQoL	HYP 100%, T2DM 96% 54 people, mean age 64 years, 48% female, mean BMI 27.3
Rodriguez-Manas et al. [52] (MID-Frail)	Europe (7 countries) 74 study sites	15 months Direct approach^a^	18 weeks PF, HRQoL	HYP 87%, T2DM 100%, HF 9% 964 people, mean age 78 years, 49% female, mean BMI 29.6
Soliman et al. [53]	Saudi Arabia n/a	5 months n/a	12 weeks DEP	DEP 100%, COPD 100% 34 people, mean age 69.7 years, 44% female, mean BMI 26.8
Gretebeck et al. [54]	USA Community centres	n/a Direct and indirect approach^a,b^	10 weeks PF	A* 36%, HYP 83%, T2DM 100% 111 people, mean age 70.5 years, 61% female, mean BMI 32.7
Campo et al. [55]	Italy Hospital	15 months Direct approach^a^	24 weeks PF, HRQoL, DEP, ANX	HYP 86%, T2DM 30%, HF 100% 235 people, mean age 76.5 years, 23% female, mean BMI 27

^a^Direct approach (potential people approached individually), ^b^indirect approach (potential people approached e.g. via news advertisement or flyers), DM* diabetes mellitus, not specified if type 1 or 2; A* arthritis, not specified which form (e.g. osteoarthritis); *DEP* depression symptoms, *HF* heart failure, *T2DM* type 2 diabetes mellitus, *COPD* chronic obstructive pulmonary disease, *KOA* knee osteoarthritis, *HOA* hip osteoarthritis, *IHD* ischemic heart disease, *BMI* body mass index (kg/m^2^), *PF* physical function, *HRQoL* health-related quality of life, *ANX* anxiety symptoms, *n/a* not applicable

### Participants characteristics

A total population of 3363 people with multimorbidity participated in the 23 RCTs. The most common diseases reported were heart failure (k=16), depression (k=15), type 2 diabetes mellitus (k=15), hypertension (k=14), chronic obstructive pulmonary disease (k=6), osteoarthritis of the knee (k=4) or hip (k=4). The number of conditions reported varied from two to seven with the most common combination being heart failure and depression [33–35, 37, 38, 44, 49] (Table 1). The mean age of the people was 65.5 (SD 8.4) years with 46% being females and the average BMI was 29.8 (SD 2.6).

### Intervention and comparator groups

The most commonly applied type of exercise therapy was aerobic exercise only (k=11) [32, 34–38, 40, 44, 49, 50, 53], followed by exercise programs combining aerobic, strengthening, balance and flexibility exercises (k=8) [33, 39, 42, 43, 45–48, 55], and Tai Chi (k=2) [41, 51] or resistance training only (k=2) [52, 54]. The duration of the exercise therapy varied from 1 to 26 weeks with a median (interquartile range (IQR)) of 12 weeks (12 to 16). Sessions per week varied from 2 to 14 with a median of 3 session IQR (3 to 3). Comparator groups varied widely and included usual care, medication, cognitive behavioural therapy, health condition education, general practitioner consultations and stretching and flexibility exercises.

### Synthesis of results

#### Recruitment rates

**Fig. 2:**
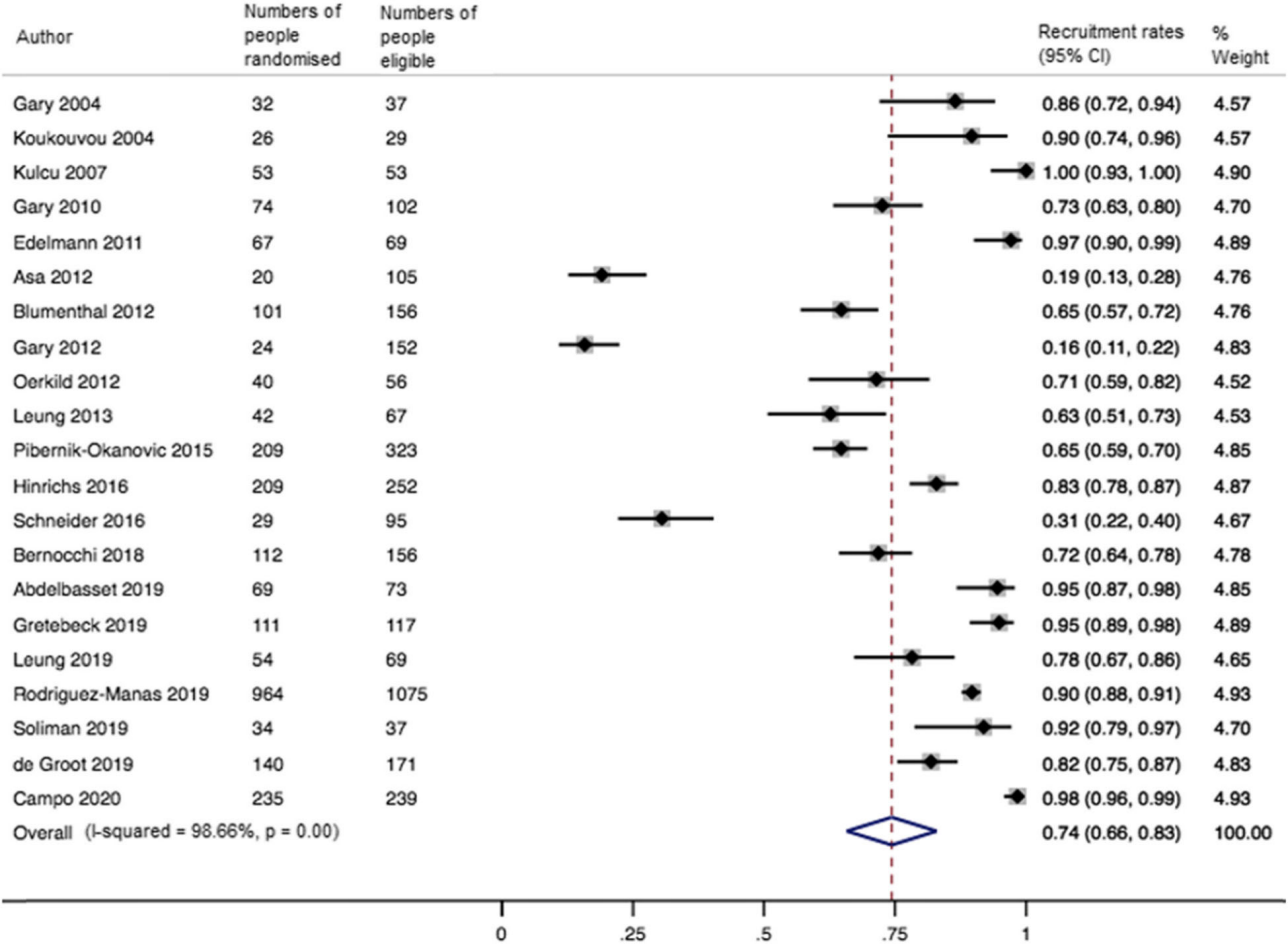
Forest plot for the recruitment rates in RCTs of exercise therapy in people with multimorbidity. 95% CI = confidence interval

The pooled recruitment rate (k=21) was 0.74 (95% CI 0.66 to 0.83; I^2^=99%) (Fig. 2). Meta-regression analyses showed no impact of recruitment strategy and trial, intervention and participant characteristics on recruitment rates (Supplementary Table 1).

#### Retention rates

**Fig. 3:**
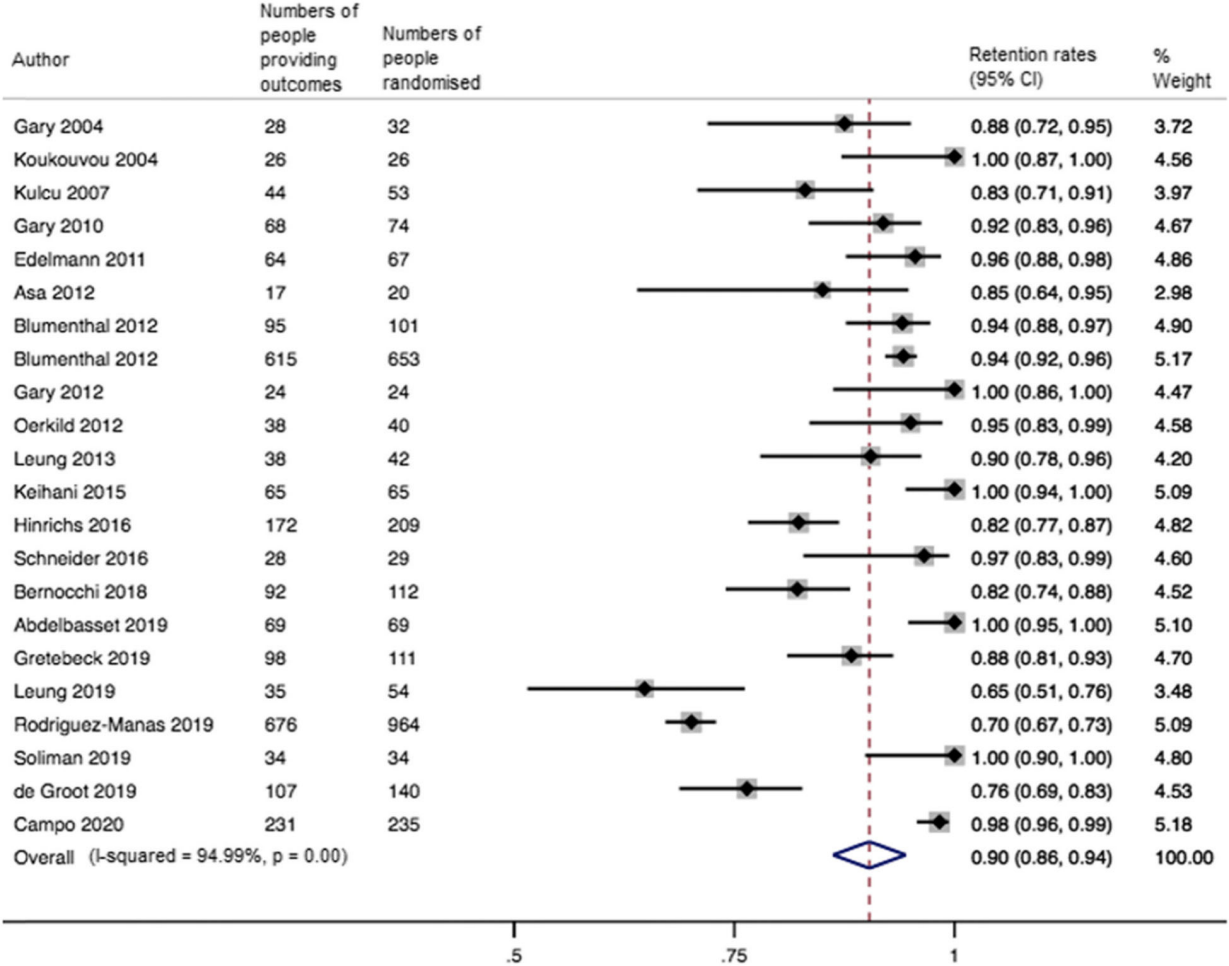
Forest plot for the retention rates in RCTs of exercise therapy in people with multimorbidity at the end of the intervention (median 12 weeks IQR 12 to 12). 95% CI = 95% confidence interval

The pooled retention rate (k=22) was 0.90 (95% CI 0.86 to 0.94; I^2^=95%) at the end of the intervention (median 12 weeks IQR 12 to 12) (Fig. 3). Meta-regression analyses showed that there was no difference in retention rates between physical and psychosocial outcomes and that increasing age (slope −0.01; 95% CI −0.01 to 0.01; Tau^2^=.006) and including a higher proportion of people with hypertension (slope −0.01; 95% CI −0.01 to −0.01; Tau^2^=.006) were associated with lower retention rates (Supplementary Table 1 and Supplementary Figures 2a and b). This suggests that for every year the age increase, the retention rates were reduced with 1%. Similarly, for each additional percentage of people with hypertension included in the study, the retention rates were reduced by 1%.

Ten studies were included in the meta-analysis at the follow-up time closest to 12 months. The pooled retention rate was 0.80 (95% CI 0.68 to 0.92; I^2^=98%).

#### Differential retention rates

**Fig. 4:**
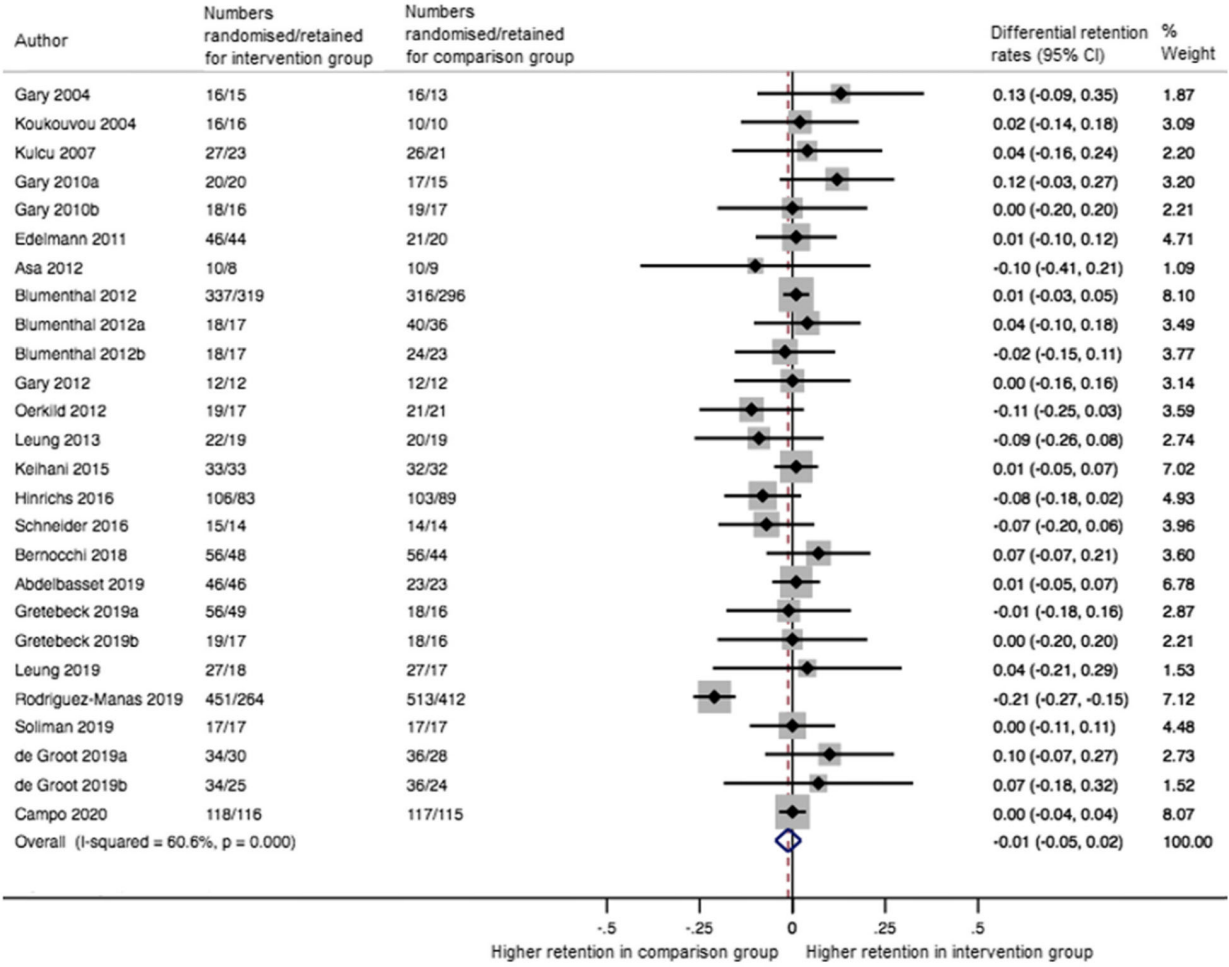
Forest plot for the differential retention rates in RCTs of exercise therapy in people with multimorbidity at the end of the intervention (median 12 weeks IQR 12 to 12). 95% CI = 95% confidence interval

The pooled differential retention rate (k=22) was −0.01 (95% CI −0.05 to 0.02; I^2^=61%) at the end of the intervention (median 12 weeks IQR 12 to 12) (Fig. 4). Meta-regression analyses showed no impact of outcome domain (physical vs. psychosocial outcomes) and intervention and comparator characteristics on differential retention rates (Supplementary Table 2).

Ten studies were included in the meta-analysis at the follow-up time closest to 12 months. The pooled differential retention was 0.01 (95% CI −0.06 to 0.08; I^2^=73%).

#### Risk of bias within studies

Overall, the risk of bias assessment showed that none of the studies was judged as having ‘low risk of bias’, 91% as having ‘some concerns’, and 9% as having ‘high risk of bias’, respectively (Supplementary Figure 1). A randomisation process with a low risk of bias was seen in 78% of the studies, and only 9% had bias due to deviations from intended interventions. The domain causing the greatest risk of bias was the measurement of the outcome as the outcome assessors in all the studies were participants filling out self-completed questionnaires, which made blinding particularly difficult.

#### Quality of the evidence

The overall quality of the evidence, including the reasons for grading the evidence, was summarised in Table 2. Overall, the quality of evidence was deemed as very low. We started the GRADE assessment from low as recommended for observational studies since although this systematic review included RCTs they did not test the effect of recruitment and retention strategies on effect estimates. Inconsistency and indirectness were the two reasons for downgrading the quality of the evidence due to the inclusion of people with depression and heart failure in most of the studies and the inability to explain the inconsistency of the estimates with meta-regression analyses.

**Table 2:** Summary of findings

**People with multimorbidity participating in exercise therapy RCTs**				
**Patient or population:** People with two or more chronic conditions **Intervention:** Exercise therapy **Comparison:** Usual care, education, or other non-exercise groups				
**Outcomes follow-up**	**Proportion (95% CI)**	**I-squared**	**Numbers of people (studies)**	**Quality of the evidence (GRADE)**
**Recruitment rates**	0.74 (0.66, 0.83)	99%	2645 (21 RCTs)	⊕ ⊖ ⊖ ⊖^a,b^ (Very low)
**Retention rates** Timepoint: end of treatment	0.90 (0.86, 0.94)	95%	3154 (22 RCTs)	⊕ ⊖ ⊖ ⊖^a,b^ (Very low)
Differential retention rates* Timepoint: end of treatment	−0.01 (−0.05, 0.02)	61%	3154 (22 RCTs)	⊕ ⊖ ⊖ ⊖^a,b^ (Very low)

*The basis of the differential retention rate is calculated by subtracting numbers providing intervention outcomes from numbers providing comparison outcomes

RCTs (randomised controlled trials), CI (95% Confidence Interval), GRADE (Grading of Recommendations Assessment, Development and Evaluation)

GRADE Working Group grades of evidence

High quality: Further research is very unlikely to change our confidence in the estimate of effect

Moderate quality: Further research is likely to have an important impact on our confidence in the estimate of effect and may change the estimate

Low quality: Further research is very likely to have an important impact on our confidence in the estimate of effect and is likely to change the estimate

Very low quality: We are very uncertain about the estimate

^a^Downgraded one due to considerable heterogeneity without a plausible explanation

^b^Downgraded one due to indirectness of people

## Discussion

This systematic review investigating recruitment and retention rates included 23 exercise therapy RCTs with the participation of more than 3300 people with multimorbidity performed in 18 countries. On average, 74% of the eligible people were randomised to RCTs. Of these, 90% provided outcomes at the end of the intervention assessment. Recruiting people with increasing age and/or with chronic hypertension plus a coexisting condition showed lower retention rates. Retention rates did not vary between the intervention groups and comparison groups.

### Recruitment rates in people with multimorbidity

Three out of four eligible people were randomised to RCTs of exercise therapy. This recruitment rate was higher (74% vs 64%) than in previous RCTs including people with multimorbidity patient-centred interventions, without an exercise component [18, 19]. However, the population in our systematic review was younger and the combination of diseases was different. Similarly, in exercise RCTs, recruitment rates were higher when including people with multimorbidity rather than when including people with single chronic conditions such as chronic obstructive pulmonary disease (54%), heart failure (41%) or depression (65%) [56–58]. Comparing with these previous findings, this suggests that for exercise therapy RCTs, eligible people with multimorbidity might be easier to recruit compared to people with a single condition. However, the indirect comparisons of the recruitment rates need to be interpreted with caution due to differences in participant characteristics and setting of interventions.

### Retention and differential rates in people with multimorbidity

Overall, nine in 10 people with multimorbidity provided end of treatment outcomes, with no difference between retaining people in the intervention and comparison groups. The overall retention rates were slightly higher (90% versus 86%) than previously reported retention rates of multimorbidity RCTs in general [18, 19]. Additionally, the rates found were similar to RCTs including people with either chronic obstructive pulmonary disease (82%), heart failure (97%) or depression (100%) [56–58], suggesting that people with multimorbidity are just as likely to provide end of treatment outcomes as people with a single disease. However, for longer-term follow-up assessment, the retention rates found diminished from 90 to 80%. This is somewhat expected and highlights the need of putting extra effort into retaining participants for long-term outcome assessments [12]. Additionally, our findings suggest that including people with increasing age and/or people with hypertension plus another coexisting condition is potentially less likely to provide end of treatment outcomes than younger people and/or people with multimorbidity consisting of other chronic conditions. Increasing age has previously been found to negatively affect attrition rates in RCTs [59]. However, while we are unsure why hypertension in this study is associated with lower retention rates, it appears that retaining people with hypertension in exercise trials is particularly challenging [60]. Finally, our findings also suggest that retention rates do not vary between the intervention and comparison groups as previously hypothesised.

### Strengths and limitations

This systematic review with meta-analyses followed a pre-specified protocol made publicly available prior to completion of the title/abstract screening and followed recommended guidelines for conducting and reporting systematic reviews [22, 24]. Additionally, authors of the included RCTs were emailed to obtain missing data, which enabled us to perform meta-regression analyses with more complete information on the trial, participant and intervention characteristics associated with recruitment, retention and differential retention.

This systematic review has some limitations. The number of RCTs included is small and from many different countries. However, this was expected since multimorbidity is a relatively new concept and to improve chances of identifying all studies available, a comprehensive search strategy developed for a previously published systematic review was used [23]. Additionally, the prevalence of people with osteoarthritis, hypertension, type 2 diabetes mellitus, depression, heart failure, ischemic heart disease and chronic obstructive pulmonary disease varied considerably across all the included RCTs. Most people randomised had either depression, heart failure or a combination of both diseases, which limits the generalizability of our findings to the whole population with multimorbidity. Furthermore, as expected by the multimorbidity definition, we found considerable heterogeneity when pooling the results, and only a few of the trial, participant and intervention characteristics were able to explain some of the heterogeneous results for the meta-analyses. Finally, reporting of both recruitment and retention strategies was inconsistent. For example, none of the included RCTs reported any form of reminders for non-responders and only one reported the use of financial incentives [46].

### Implication for future research

The optimal strategy for recruiting and retaining people with multimorbidity in exercise RCTs remains unclear, partially due to the inconsistent reporting of recruitment and retention strategies in existing trials. Therefore, it is important that future exercise RCTs including people with multimorbidity should describe the strategies used in a detailed and transparent way and, if feasible and available, use existing strategies proven to be effective. For example, using patient and public involvement may increase recruitment and retention rates as it helps to ensure that the research focuses on issues relevant to patients and the public [61]. Additionally, the use of an open trial design and telephone reminders for non-responders to postal interventions to improve recruitment [62] and the use of monetary incentives to increase retention [63] has proven to be effective. Finally, in order to enhance the evidence base for strategies that are useful to improve recruitment and retention rates, future exercise RCTs should consider testing the effect of different recruitment and retention strategies by performing Studies Within A Trial [64], which are self-contained studies embedded within a trial aimed at evaluating or exploring different ways of delivering or organising a specific trial process.

## Conclusions

Three in four eligible people with multimorbidity were randomised to exercise therapy RCTs, of which nine out of 10 provided end of treatment outcomes, with no difference seen between the intervention and comparison groups. Enrolling people with increasing age and/or with chronic hypertension plus a coexisting condition could appear to lead to lower retention rates. However, the results must be interpreted with caution due to large differences between the included studies.

## Supplementary Information

**Additional file 1.** PRISMA 2009 Checklist.

**Additional file 2: Supplementary Figure 1.** ‘Risk of bias’ summary shown as percentage of 23 individual ‘Risk of bias’ items for each included study**. Supplementary Table 1.** Impact of covariates on recruitment and retention rates. **Supplementary Table 2.** Impact of covariates on differential retention rates. **Supplementary Figure 2.** Bubble plot for the impact of age (a) and proportion of included participants with hypertension (b) on retention rates.

## Data Availability

The dataset and statistical script necessary to reproduce the analyses presented in this systematic review has been made available online at the Open Science Framework page of the MOBILIZE project [26].

## Abbreviations

RCTs: Randomised controlled trials
HRQoL: Health-related quality of life
PRISMA: Preferred Reporting Items for Systematic Reviews and Meta-analyses
MEDLINE: Medical Literature Analysis and Retrieval System Online
CENTRAL: Cochrane Central Register of Controlled Trials
ICTRP: International Clinical Trials Registry Platform
WoS: Web of Science
TIAB: Title/Abstract
MeSH: Medical Subject Heading
GRADE: Grading of Recommendations Assessment, Development and Evaluation
SD: Standard deviation
SE: Standard error
95% CI: 95% confidence interval
BMI: Body mass index
SES: Socio economic status
IQR: Interquartile range
