# Supplementary material for: Recruitment and retention rates in randomised controlled trials of exercise therapy in people with multimorbidity: a systematic review and meta-analysis
Source: Trials. 2021 Jun 14;22:396. doi: 10.1186/s13063-021-05346-x (PMC8204443; doi:10.1186/s13063-021-05346-x)

# Figures, tables, and additional files


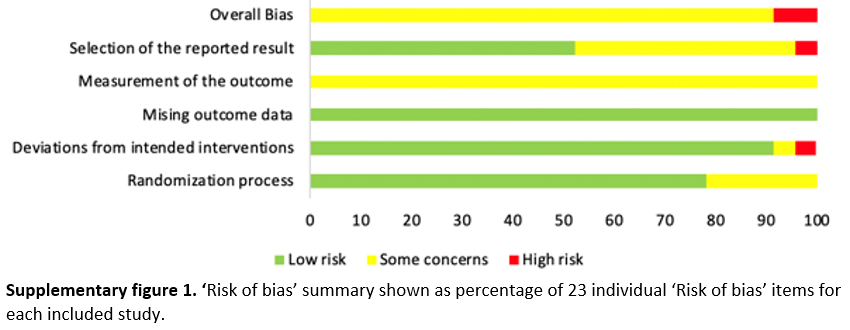


Supplementary table 1. Impact of covariates on recruitment and retention rates

| **Variable** | **k_RCTs** | **Recruitment rates proportion** | **95% CI** | **Tau^2^** | **k_RCTs** | **Retention rates proportion** | **95% CI** | **Tau^2^** |
| --- | --- | --- | --- | --- | --- | --- | --- | --- |
|  | 21 | 0.74 | 0.63, 0.86 | .061 | 22 | 0.90 | 0.86, 0.95 | .008 |
| **Participant characteristics** | **k_RCTs** | **Coefficient** | **95% CI** | **Tau^2^** | **k_RCTs** | **Coefficient** | **95% CI** | **Tau^2^** |
| Age (years) | 20 | 0.01 | -0.01, 0.02 | .065 | 21 | -0.01 | -0.01, 0.01 | .006 |
| Proportion of women | 20 | -0.01 | -0.01, 0.01 | .067 | 21 | -0.01 | -0.01, 0.01 | .008 |
| BMI (kilogram) | 18 | -0.04 | -0.09, 0.02 | .063 | 19 | 0.01 | -0.02, 0.02 | .009 |
| Low SES (no vs. yes) | 12 | -0.13 | -0.53, 0.27 | .070 | 13 | -0.04 | -0.21, 0.13 | .011 |
| Proportion of people with hypertension | 14 | 0.01 | -0.01, 0.01 | .048 | 15 | -0.01 | -0.01, -0.01 | .006 |
| Proportion of people with type 2 diabetes mellitus | 15 | -0.01 | -0.01, 0.01 | .075 | 15 | -0.01 | -0.01, -0.01 | .006 |
| Proportion of people with depression | 13 | 0.01 | -0.01, 0.01 | .070 | 14 | 0.01 | -0.01, 0.01 | .004 |
| Depression disease severity | 11 | -0.05 | -0.17, 0.08 | .078 | 12 | -0.01 | -0.04, 0.02 | .004 |
| Proportion of people with heart failure | 15 | -0.01 | -0.01, 0.01 | .072 | 17 | 0.01 | 0.01, 0.01 | .002 |
| **Intervention/comparator characteristics** |  |  |  |  |  |  |  |  |
| Type of intervention used:   - Aerobic vs. tai chi - Aerobic vs. mixed | 21 | -0.05 -0.04 | -0.50, 0.39 -0.31, 0.23 | .070 | 22 | -0.13 0.01 | -0.28, 0.02 -0.07, 0.09 | .006 |
| Type of comparator used:   - Usual care vs. education - Usual care vs. others | 21 | 0.01 -0.17 | -0.28, 0.30 -0.43, 0.09 | .060 | 22 | -0.04 -0.04 | -0.15, 0.08 -0.14, 0.06 | .008 |
| Frequency of intervention sessions | 20 | -0.01 | -0.10, 0.08 | .067 | 21 | 0.01 | -0.03, 0.04 | .007 |
| Mode of delivery:   - 1 to 1 vs. group-based - 1 to 1 vs. self-help | 19 | -0.09 0.06 | -0.40, 0.22 -0.29, 0.41 | .067 | 19 | -0.07 -0.01 | -0.18, 0.03 -0.12, 0.11 | .007 |
| Intervention setting used:   - Home vs. clinic - Home vs. combination | 20 | -0.03 0.07 | -0.29, 0.24 -0.38, 0.52 | .071 | 20 | -0.06 -0.14 | -0.15, 0.03 -0.29, 0.01 | .007 |
| Length of intervention (weeks) | 21 | 0.01 | -0.01, 0.01 | .064 | 22 | 0.01 | -0.01, 0.01 | .009 |
| Supervision (supervised vs. partially supervised) | 21 | -0.05 | -0.28, 0.19 | .064 | 22 | -0.01 | -0.11, 0.08 | .009 |
| Adherence to intervention (number of sessions attended of total number of sessions available) | n/a |  |  |  | 13 | -0.01 | -0.01, 0.01 | .010 |
| Session intensity (low to moderate vs. moderate) | 20 | -0.01 | -0.28, 0.26 | .068 | 20 | 0.03 | -0.08, 0.14 | .009 |
| **Study characteristics** |  |  |  |  |  |  |  |  |
| Size of included studies (people randomised) | n/a |  |  |  | 22 | -0.01 | -0.01, 0.01 | .007 |
| Recruitment setting (hospital vs. outpatient) | 19 | -0.10 | -0.33, 0.12 | .049 | 19 | -0.08 | -0.17, 0.02 | .008 |
| Recruitment strategy approach (direct or indirect vs. combination) | 15 | 0.01 | -0.27, 0.28 | .058 | 16 | 0.02 | -0.10, 0.14 | .011 |
| Recruitment length (months) | 18 | -0.01 | -0.01, 0.01 | .049 | 18 | -0.01 | -0.01, 0.01 | .010 |
| Reason for dropout (no vs. yes) | n/a |  |  |  | 22 | 0.02 | -0.08, 0.11 | .009 |
| Risk of bias (low* vs. some concerns or high) | 21 | 0.03 | -0.21, 0.26 | .064 | 22 | -0.02 | -0.11, 0.07 | .009 |
| Type of outcome assessment (only objectively measured vs. only patient-reported outcomes) | n/a |  |  |  | 10 | 0.12 | -0.02, 0.25 | .006 |
| Number of people assessed for eligibility | 21 | -0.01 | -0.01, 0.01 | .062 | n/a |  |  |  |
| Number of people assessed for eligibility in person | 11 | 0.01 | -0.01, 0.01 | .042 | n/a |  |  |  |
| Pre-screening of people (no vs. yes) | 13 | -0.08 | -0.42, 0.25 | .062 | n/a |  |  |  |
| k_RCTs (number of randomised controlled trails), recruitment rates proportion (proportion of people randomised/proportion of people eligible), retention rates proportion (proportion of people providing the outcomes of interest/proportion randomised), 95% CI (95% Confidence Interval), Tau^2^ (between study variation), coefficient (overall percentage of recruitment or retention rate proportion), BMI (Body mass index), low SES (when majority of people are described as having low education levels, low income, being unemployed or sample otherwise labelled as `low SES`), vs. (versus), n/a (not applicable), *Since people enrolled in exercise trials cannot be blinded to the exercise interventions, studies with Low risk of Bias are the studies judged as Low Risk of Bias in all domains but ´measurement of the outcome´. Studies judged as some concerns have been judged at Some ´Concerns for at least one more item then ´measurement of the outcome´ | | | | | | | | |

Supplementary table 2. Impact of covariates on differential retention rates

| **Variable** | **n_observations** | **Differential retention rates proportion** | **95% CI** | **Tau^2^** |
| --- | --- | --- | --- | --- |
|  | 26 | -0.01 | -0.05, 0.03 | .0035 |
| **Intervention/comparator characteristics** | **n_observations** | **Coefficient** | **95% CI** | **Tau^2^** |
| Type of comparator used:   - Usual care vs. education - Usual care vs. others | 26 | 0.01 0.01 | -0.11, 0.12 -0.08, 0.10 | .0040 |
| Type of intervention used:   - Aerobic vs tai chi - Aerobic vs. mixed | 26 | -0.06 -0.02 | -0.21, 0.09 -0.06, 0.02 | 0 |
| Difference in number of sessions between intervention and comparison groups | 22 | 0.01 | -0.01, 0.01 | .0039 |
| Intervention mode of delivery:   - 1 to 1 vs. group-based - 1 to 1 vs. self-help | 22 | -0.07 0.01 | -0.16, 0.03 -0.09, 0.11 | .0030 |
| Intervention setting:   - Home vs. clinic - Home vs. combination | 23 | -0.06 -0.02 | -0.15, 0.03 -0.15, 0.12 | .0039 |
| Intervention supervision:  Supervised vs partially supervised | 26 | 0.03 | -0.05, 0.10 | .0036 |
| n_observations (number of observations), differential retention rates proportion (difference in proportion of people providing physical and/or psychosocial outcomes in the intervention and comparator group), 95% CI (95% Confidence Interval), Tau^2^ (between study variation), coefficient (overall percentage of differential retention rate proportion), vs. (versus) | | | | |


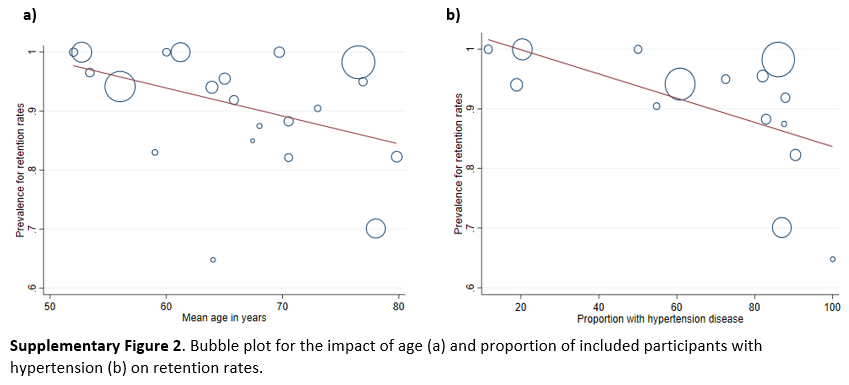

Supplement: Supplementary file 2 — Additional file 2: Supplementary Figure 1. ‘Risk of bias’ summary shown as percentage of 23 individual ‘Risk of bias’ items for each included study. Supplementary Table 1. Impact of covariates on recruitment and retention rates. Supplementary Table 2. Impact of covariates on differential retention rates. Supplementary Figure 2. Bubble plot for the impact of age (a) and proportion of included participants with hypertension (b) on retention rates. [file 13063_2021_5346_MOESM2_ESM.docx]
